# Supplementary material for: Exploring the classical and numerical Delboeuf illusion: the impact of transcranial alternating current stimulation on magnitude processing
Source: PeerJ. 2025 Mar 10;13:e19064. doi: 10.7717/peerj.19064 (PMC11905914; doi:10.7717/peerj.19064)
Supplement: Supplemental Information 2 — The electric field distribution, individual and group performance, additional figures of the performances in the two discrimination tasks and all the post-hoc comparisons of all the GLMMs. [file peerj-13-19064-s002.docx]

**Supplementary Material**

**Figure S1.** Electric field distribution. Figure created using SimNIBS 4, which allows for realistic calculations of the electric field induced by transcranial electrical stimulation. Specifically, the present protocol targeted the right and left parietal cortex, which are approximated by electrode positions P3 and P4 of the 10-10 EEG system, using the Ernie model in the SimNIBS software (Saturnino, Madsen, & Thielscher, 2019).


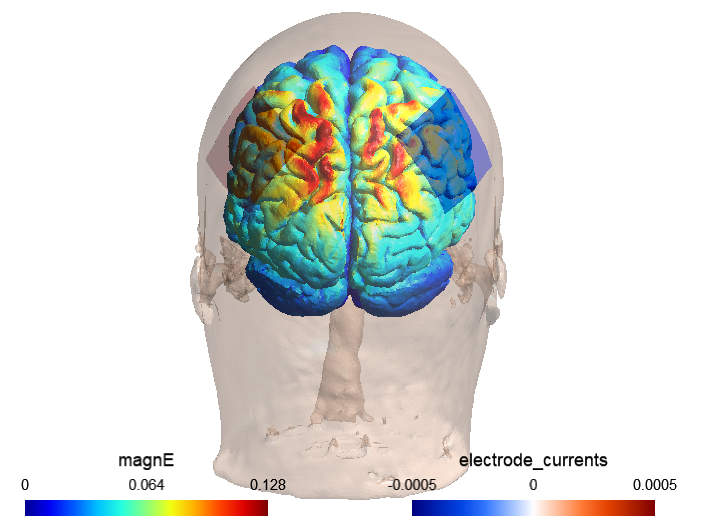


[Saturnino, G. B., Siebner, H. R., Thielscher, A., & Madsen, K. H. (2019). Accessibility of cortical regions to focal TES: Dependence on spatial position, safety, and practical constraints. NeuroImage, 203, 116183.](https://doi.org/10.1016/j.neuroimage.2019.116183)

**Table S1.** Individual and group performance (small, large, congruent, incongruent trials: frequency of choices for the larger numerosity and area; Delboeuf illusion: frequency of choices for the expected larger numerosity, i.e., presented in the small background). Statistics were calculated with binomial tests for individual analyses and with one-sample *t* tests or Wilcoxon-signed rank tests for group analyses. Asterisks (*) denote a significant departure from chance level (0.5).

|  |  |  | **Continuous quantity discrimination** | | | | | **Numerical discrimination** | | | | |  |  |  |
| --- | --- | --- | --- | --- | --- | --- | --- | --- | --- | --- | --- | --- | --- | --- | --- |
| **Subject** | **Age** | **Gender** | **Small** | **Large** | **Congruent** | **Incongruent** | **Delboeuf illusion** | **Small** | **Large** | **Congruent** | **Incongruent** | **Delboeuf illusion** | **tAcs** |  |  |
| 1 | 24 | F | 50/60  *p*<0.001* | 56/60  *p*<0.001* | 1/59  *p*<0.001* | 60/60  *p*<0.001* | 60/60  *p*<0.001* | 54/59  *p*<0.001* | 53/60  *p*<0.001* | 49/60  *p*<0.001* | 58/60  *p*<0.001* | 40/60  *p=*0.013* | Sham |  |  |
|  |  |  | 51/59  *p*<0.001* | 55/60  *p*<0.001* | 1/59  *p*<0.001* | 60/60  *p*<0.001* | 60/60  *p*<0.001* | 46/56  *p*<0.001* | 51/60  *p*<0.001* | 46/58  *p*<0.001* | 53/60  *p*<0.001* | 43/60  *p=*0.001* | 7 Hz |  |  |
|  |  |  | 48/60  *p*<0.001* | 48/57  *p*<0.001* | 4/60  *p*<0.001* | 60/60  *p*<0.001* | 60/60  *p*<0.001* | 46/59  *p*<0.001* | 48/57  *p*<0.001* | 45/60  *p*<0.001* | 51/58  *p*<0.001* | 30/59  *p=*1.000 | 18 Hz |  |  |
| 2 | 30 | M | 37/58  *p*=0.048* | 41/60  *p*=0.006* | 7/60  *p*<0.001* | 59/60  *p*<0.001* | 59/60  *p*<0.001* | 37/54  *p*=0.009* | 33/52  *p*=0.070 | 13/54  *p*<0.001* | 49/55  *p*<0.001* | 39/53  *p*<0.001* | Sham |  |  |
|  |  |  | 18/28  *p*=0.185 | 24/37  *p*=0.099 | 12/41  *p*=0.012* | 30/32  *p*<0.001* | 28/31  *p*<0.001* | 37/54  *p=*0.009* | 40/52  *p*<0.001* | 33/56  *p*=0.229 | 40/54  *p*<0.001* | 30/52  *p=*0.332 | 7 Hz |  |  |
|  |  |  | 48/58  *p*<0.001* | 38/58  *p*=0.025* | 12/59  *p*<0.001* | 58/60  *p*<0.001* | 55/59  *p*<0.001* | 42/57  *p*<0.001* | 35/59  *p*=0.193 | 24/57  *p*=0.289 | 48/57  *p*<0.001* | 37/55  *p=*0.015* | 18 Hz |  |  |
| 3 | 22 | F | 45/59  *p*<0.001* | 49/59  *p*<0.001* | 10/58  *p*<0.001* | 56/58  *p*<0.001* | 52/58  *p*<0.001* | 32/58  *p*=0.512 | 30/55  *p*=0.590 | 8/57  *p*<0.001* | 49/55  *p*<0.001* | 36/59  *p*=0.117 | Sham |  |  |
|  |  |  | 44/57  *p*<0.001* | 39/48  *p*<0.001* | 8/57  *p*<0.001* | 53/56  *p*<0.001* | 52/56  *p*<0.001* | 34/55  *p=*0.105 | 38/55  *p*=0.006* | 17/55  *p*=0.006* | 47/57  *p*<0.001* | 41/57  *p*=0.001* | 7 Hz |  |  |
|  |  |  | 34/49  *p*=0.009* | 34/42  *p*<0.001* | 14/55  *p*<0.001* | 54/55  *p*<0.001* | 40/49  *p*<0.001* | 32/51  *p*=0.092 | 23/40  *p*=0.430 | 14/51  *p*=0.001* | 39/43  *p*<0.001* | 32/43  *p=*0.002* | 18 Hz |  |  |
| 4 | 22 | F | 31/60  *p*=0.897 | 32/59  *p*=0.603 | 16/58  *p*<0.001* | 45/60  *p*<0.001* | 43/59  *p*<0.001* | 29/60  *p*=0.897 | 37/59  *p*=0.067 | 31/59  *p*=0.795 | 32/60  *p*=0.699 | 32/60  *p*=0.699 | Sham |  |  |
|  |  |  | 35/59  *p*=0.193 | 33/60  *p*=0.519 | 16/59  *p*<0.001* | 52/60  *p*<0.001* | 49/60  *p*<0.001* | 31/60  *p=*0.897 | 31/60  *p=*0.897 | 46/60  *p<*0.001* | 17/60  *p*=0.001* | 17/60  *p*=0.001* | 7 Hz |  |  |
|  |  |  | 34/59  *p*=0.298 | 36/60  *p=*0.155* | 25/60  *p*=0.245 | 49/58  *p*<0.001* | 47/59  *p*<0.001* | 28/60  *p*=0.699 | 38/60  *p*=0.052 | 41/59  *p*=0.004* | 23/60  *p*=0.092 | 21/60  *p=*0.027* | 18 Hz |  |  |
| 5 | 24 | M | 31/59  *p*=0.795 | 31/60  *p=*0.897 | 31/60  *p=*0.897 | 37/60  *p*=0.092 | 33/59  *p*=0.435 | 35/59  *p*=0.193 | 43/59  *p*=0.001* | 29/58  *p*=1.000 | 37/59  *p*=0.067 | 28/60  *p*=0.699 | Sham |  |  |
|  |  |  | 27/58  *p*=0.694 | 31/58  *p*=0.694 | 28/59  *p*=0.795 | 26/58  *p*=0.512 | 28/59  *p*=0.795 | 33/57  *p=*0.289 | 27/60  *p=*0.519 | 31/60  *p*=0.897 | 32/59  *p*=0.603 | 34/58  *p*=0.237 | 7 Hz |  |  |
|  |  |  | 31/60  *p*=0.897 | 29/56  *p=*0.894 | 34/58  *p*=0.237 | 34/58  *p*=0.237 | 27/53  *p*=1.000 | 36/58  *p*=0.087 | 37/56  *p*=0.022* | 26/58  *p*=0.512 | 39/57  *p*=0.008* | 30/56  *p=*0.689 | 18 Hz |  |  |
| 6 | 23 | F | 36/60  *p*=0.155 | 30/55  *p=*0.590 | 17/56  *p=*0.005* | 41/60  *p*=0.006* | 37/57  *p*=0.033* | 35/58  *p*=0.148 | 31/58  *p*=0.694 | 33/57  *p*=0.289 | 38/58  *p*=0.025* | 29/57  *p*=1.000 | Sham |  |  |
|  |  |  | 35/58  *p*=0.148 | 34/60  *p*=0.366 | 27/59  *p*=0.603 | 39/60  *p*=0.027* | 41/59  *p*=0.004* | 33/59  *p=*0.559 | 31/59  *p=*0.795 | 36/59  *p*=0.117 | 27/59  *p*=0.603 | 29/60  *p*=0.897 | 7 Hz |  |  |
|  |  |  | 31/60  *p*=0.897 | 39/58  *p=*0.012* | 26/56  *p*=0.689 | 46/57  *p*<0.001* | 42/59  *p*=0.002* | 38/60  *p*=0.052 | 37/59  *p*=0.067 | 27/59  *p*=0.603 | 30/60  *p*=1.000 | 34/60  *p=*0.366 | 18 Hz |  |  |
| 7 | 24 | F | 36/57  *p*=0.063 | 39/58  *p*=0.012* | 21/59  *p*=0.036* | 56/59  *p*<0.001* | 51/59  *p*<0.001* | 47/60  *p*<0.001* | 53/60  *p*<0.001* | 51/59  *p*<0.001* | 43/60  *p*=0.001* | 19/60  *p*<0.001* | Sham |  |  |
|  |  |  | 45/58  *p*<0.001* | 36/51  *p*=0.005* | 27/54  *p*=1.000 | 54/56  *p*<0.001* | 42/52  *p*<0.001* | 48/58  *p*<0.001* | 43/58  *p*<0.001* | 54/59  *p*<0.001* | 24/60  *p*=0.155 | 14/59  *p*<0.001* | 7 Hz |  |  |
|  |  |  | 44/59  *p*<0.001* | 34/58  *p=*0.237 | 26/59  *p*=0.435 | 56/60  *p*<0.001* | 51/60  *p*<0.001* | 50/59  *p*<0.001* | 48/59  *p*<0.001* | 59/60  *p*<0.001* | 35/60  *p*=0.245 | 16/59  *p*<0.001* | 18 Hz |  |  |
| 8 | 22 | F | 50/59  *p*<0.001* | 51/60  *p*<0.001* | 22/60  *p*=0.052 | 57/60  *p*<0.001* | 56/60  *p*<0.001* | 34/60  *p*=0.366 | 39/59  *p*=0.018* | 22/60  *p*=0.052 | 40/60  *p*=0.013* | 29/60  *p*=0.897 | Sham |  |  |
|  |  |  | 37/60  *p*=0.092 | 36/60  *p*=0.115 | 23/60  *p*=0.092 | 44/59  *p*<0.001* | 41/57  *p*=0.001* | 38/58  *p*=0.025* | 43/60  *p*=0.001* | 25/60  *p*=0.245 | 55/60  *p*<0.001* | 41/59  *p*=0.004* | 7 Hz |  |  |
|  |  |  | 45/60  *p*<0.001* | 42/60  *p=*0.003* | 30/60  *p*=1.000 | 52/60  *p*<0.001* | 40/60  *p*=0.013* | 40/60  *p*=0.013* | 50/60  *p*<0.001* | 20/60  *p*=0.013* | 56/60  *p*<0.001* | 48/60  *p*<0.001* | 18 Hz |  |  |
| 9 | 25 | F | 43/60  *p*<0.001* | 40/59  *p*=0.009* | 22/60  *p*=0.052 | 53/60  *p*<0.001* | 47/60  *p*<0.001* | 37/60  *p*=0.092 | 30/59  *p*=1.000 | 47/59  *p*<0.001* | 37/59  *p*=0.067 | 32/59  *p*=0.603 | Sham |  |  |
|  |  |  | 30/59  *p*=1.000 | 29/59  *p*=1.000 | 7/60  *p*<0.001* | 54/58  *p*<0.001* | 49/60  *p*<0.001* | 33/60  *p*=0.519 | 35/60  *p*=0.245 | 25/60  *p*=0.245 | 34/60  *p*=0.366 | 41/60  *p*=0.006* | 7 Hz |  |  |
|  |  |  | 46/59  *p*<0.001* | 43/60  *p=*0.001* | 9/60  *p*<0.001* | 58/59  *p*<0.001* | 54/60  *p*<0.001* | 33/60  *p*=0.519 | 40/60  *p*=0.013* | 48/60  *p*<0.001* | 43/60  *p=*0.001* | 37/60  *p*=0.092 | 18 Hz |  |  |
| 10 | 24 | F | 35/60  *p*=0.245 | 33/59  *p*=0.435 | 19/60  *p*=0.006* | 37/60  *p*=0.092 | 46/60  *p*<0.001* | 31/60  *p*=0.897 | 35/59  *p*=0.193 | 17/59  *p*=0.002* | 46/60  *p*<0.001* | 41/60  *p*=0.006* | Sham |  |  |
|  |  |  | 38/60  *p*=0.052 | 39/59  *p*=0.018* | 13/60  *p*<0.001* | 55/59  *p*<0.001* | 52/58  *p*<0.001* | 33/60  *p*=0.519 | 35/60  *p*=0.245 | 18/60  *p*=0.003* | 50/59  *p*<0.001* | 51/60  *p*<0.001* | 7 Hz |  |  |
|  |  |  | 44/60  *p*<0.001* | 39/60  *p=*0.027* | 20/60  *p*=0.013* | 45/60  *p*<0.001* | 48/59  *p*<0.001* | 38/60  *p*=0.052 | 33/60  *p*=0.519 | 26/59  *p*=0.435 | 43/59  *p=*0.001* | 38/59  *p*=0.036* | 18 Hz |  |  |
| 11 | 24 | F | 29/58  *p*=1.000 | 33/58  *p*=0.358 | 33/57  *p*=0.289 | 29/59  *p*=1.000 | 26/59  *p*=0.435 | 37/60  *p*=0.092 | 42/59  *p*=0.002* | 40/59  *p*=0.009* | 16/60  *p*<0.001* | 24/60  *p*=0.155 | Sham |  |  |
|  |  |  | 38/60  *p*=0.052 | 37/58  *p*=0.048* | 43/57  *p*<0.001* | 28/58  *p*=0.896 | 52/58  *p*<0.001* | 32/60  *p*=0.699 | 38/60  *p*=0.052 | 53/59  *p*<0.001* | 21/60  *p*=0.027* | 24/60  *p*=0.155 | 7 Hz |  |  |
|  |  |  | 26/59  *p=*0.435 | 33/59  *p=*0.435 | 31/58  *p*=0.694 | 30/59  *p*=1.000 | 33/59  *p*=0.435 | 27/56  *p*=0.894 | 30/58  *p*=0.896 | 34/60  *p*=0.366 | 24/56  *p=*0.350 | 27/58  *p*=0.694 | 18 Hz |  |  |
| 12 | 23 | M | 43/58  *p*<0.001* | 36/54  *p*=0.020* | 44/54  *p*<0.001* | 41/57  *p*=0.001* | 25/57  *p*=0.427 | 30/58  *p*=0.896 | 40/59  *p*=0.009* | 49/60  *p*<0.001* | 20/59  *p*=0.018* | 19/57  *p*=0.016* | Sham |  |  |
|  |  |  | 40/59  *p*=0.009* | 42/60  *p*=0.003* | 51/60  *p*<0.001* | 31/60  *p*=0.897 | 19/58  *p*=0.012* | 33/60  *p*=0.519 | 45/59  *p*<0.001* | 53/59  *p*<0.001* | 13/59  *p*<0.001* | 11/60  *p*<0.001* | 7 Hz |  |  |
|  |  |  | 39/59  *p=*0.018* | 47/58  *p*<0.001* | 52/58  *p*<0.001* | 23/59  *p*=0.117 | 15/60  *p*<0.001* | 42/60  *p*=0.003* | 47/60  *p*<0.001* | 9/60  *p*<0.001* | 54/59  *p*<0.001* | 11/60  *p*<0.001* | 18 Hz |  |  |
| 13 | 22 | F | 51/60  *p*<0.001* | 50/60  *p*<0.001* | 0/60  *p*<0.001* | 59/59  *p*=0.001* | 60/60  *p*=0.427 | 28/60  *p*=0.699 | 33/60  *p*=0.519 | 5/60  *p*<0.001* | 58/59  *p*<0.001* | 54/60  *p*<0.001* | Sham |  |  |
|  |  |  | 39/60  *p*=0.027* | 42/60  *p*=0.003* | 4/60  *p*<0.001* | 59/59  *p*<0.001* | 57/60  *p*<0.001* | 33/59  *p*=0.435 | 35/60  *p*=0.245 | 11/59  *p*<0.001* | 50/60  *p*<0.001* | 48/60  *p*<0.001* | 7 Hz |  |  |
|  |  |  | 47/59  *p*<0.001* | 52/59  *p*<0.001* | 1/60  *p*<0.001* | 59/60  *p*<0.001* | 58/59  *p*<0.001* | 36/58  *p*=0.087 | 32/59  *p*=0.603 | 3/59  *p*<0.001* | 57/60  *p*<0.001* | 51/59  *p*<0.001* | 18 Hz |  |  |
| 14 | 23 | F | 50/60  *p*<0.001* | 44/59  *p*<0.001* | 10/60  *p*<0.001* | 60/60  *p*<0.001* | 56/60  *p*<0.001* | 31/60  *p*=0.897 | 28/59  *p*=0.795 | 5/60  *p*<0.001* | 57/60  *p*<0.001* | 47/60  *p*<0.001* | Sham |  |  |
|  |  |  | 44/60  *p*<0.001* | 42/58  *p*=0.001* | 12/60  *p*<0.001* | 53/60  *p*<0.001* | 55/59  *p*<0.001* | 35/57  *p*=0.111 | 34/55  *p*=0.105 | 22/58  *p*=0.087 | 44/58  *p*<0.001* | 39/55  *p*=0.003* | 7 Hz |  |  |
|  |  |  | 40/60  *p*=0.013* | 48/60  *p*<0.001* | 6/59  *p*<0.001* | 60/60  *p*<0.001* | 56/60  *p*<0.001* | 27/60  *p*=0.519 | 34/59  *p*=0.298 | 8/60  *p*<0.001* | 56/60  *p*<0.001* | 51/60  *p*<0.001* | 18 Hz |  |  |
| 15 | 23 | F | 40/59  *p*=0.009* | 32/59  *p*=0.603 | 5/59  *p*<0.001* | 58/60  *p*<0.001* | 60/60  *p*<0.001* | 47/60  *p*<0.001* | 48/58  *p*<0.001* | 57/58  *p*<0.001* | 17/59  *p*=0.002* | 13/60  *p*<0.001* | Sham |  |  |
|  |  |  | 43/59  *p*=0.001* | 35/57  *p*=0.111 | 4/59  *p*<0.001* | 59/60  *p*<0.001* | 57/59  *p*<0.001* | 45/59  *p*<0.001* | 49/57  *p*<0.001* | 54/59  *p*<0.001* | 34/59  *p*<0.001* | 12/60  *p*<0.001* | 7 Hz |  |  |
|  |  |  | 44/58  *p*<0.001* | 48/60  *p*<0.001* | 6/56  *p*<0.001* | 56/59  *p*<0.001* | 57/60  *p*<0.001* | 38/55  *p*=0.006* | 36/56  *p*=0.044* | 47/57  *p*<0.001* | 25/56  *p*=0.504 | 21/55  *p*=0.105 | 18 Hz |  |  |
| 16 | 25 | F | 44/58  *p*<0.001* | 38/49  *p*<0.001* | 14/59  *p*<0.001* | 59/59  *p*<0.001* | 56/60  *p*<0.001* | 41/57  *p*=0.001* | 47/59  *p*<0.001* | 51/60  *p*<0.001* | 35/58  *p*=0.148 | 22/60  *p*=0.052 | Sham |  |  |
|  |  |  | 44/58  *p*<0.001* | 44/58  *p*<0.001* | 9/60  *p*<0.001* | 60/60  *p*<0.001* | 56/60  *p*<0.001* | 39/58  *p*=0.012* | 39/60  *p*=0.027* | 36/58  *p*=0.087 | 45/59  *p*<0.001* | 30/58  *p*=0.896 | 7 Hz |  |  |
|  |  |  | 44/60  *p*<0.001* | 46/58  *p*<0.001* | 5/59  *p*<0.001* | 60/60  *p*<0.001* | 56/60  *p*<0.001* | 48/60  *p*<0.001* | 45/57  *p*<0.001* | 43/59  *p*=0.001* | 39/57  *p*=0.008* | 28/59  *p*=0.795 | 18 Hz |  |  |
| 17 | 24 | M | 52/56  *p*<0.001* | 49/54  *p*<0.001* | 22/58  *p*=0.087 | 56/59  *p*<0.001* | 51/58  *p*<0.001* | 28/45  *p*=0.135 | 34/40  *p*<0.001* | 37/46  *p*<0.001* | 24/41  *p*=0.349 | 15/37  *p*=0.324 | Sham |  |  |
|  |  |  | 53/60  *p*<0.001* | 56/59  *p*<0.001* | 16/59  *p*=0.001* | 60/60  *p*<0.001* | 56/60  *p*<0.001* | 37/60  *p*=0.092 | 49/60  *p*<0.001* | 49/60  *p*<0.001* | 30/60  *p*=1.000 | 31/59  *p*=0.795 | 7 Hz |  |  |
|  |  |  | 48/60  *p*<0.001* | 48/59  *p*<0.001* | 22/59  *p*=0.067 | 59/59  *p*<0.001* | 58/59  *p*<0.001* | 35/59  *p*=0.193 | 38/60  *p*=0.052 | 45/60  *p*<0.001* | 24/60  *p*=0.155 | 22/60  *p*=0.052 | 18 Hz |  |  |
| 18 | 35 | F | 43/60  *p*=0.001* | 44/60  *p*<0.001* | 28/59  *p*=0.795 | 50/60  *p*<0.001* | 35/60  *p*=0.245 | 31/60  *p*=0.897 | 33/60  *p*=0.519 | 27/60  *p*=0.519 | 45/60  *p*<0.001* | 36/59  *p*=0.117 | Sham |  |  |
|  |  |  | 35/60  *p*=0.245 | 35/60  *p*=0.245 | 2/60  *p*<0.001* | 59/60  *p*<0.001* | 57/60  *p*<0.001* | 31/60  *p*=0.897 | 35/60  *p*=0.245 | 15/60  *p*<0.001* | 49/60  *p*<0.001* | 52/60  *p*<0.001* | 7 Hz |  |  |
|  |  |  | 41/60  *p*=0.006* | 34/58  *p*=0.237 | 11/60  *p*<0.001* | 49/59  *p*<0.001* | 51/59  *p*<0.001* | 38/59  *p*=0.036 | 31/60  *p*=0.897 | 12/59  *p*<0.001* | 51/60  *p*<0.001* | 43/60  *p*=0.001* | 18 Hz |  |  |
| 19 | 22 | F | 32/60  *p*=0.699 | 33/60  *p*=0.519 | 20/59  *p*=0.018* | 42/57  *p*<0.001* | 39/58  *p*=0.012* | 32/60  *p*=0.699 | 33/60  *p*=0.519 | 28/59  *p*=0.795 | 42/60  *p*=0.003* | 24/59  *p*=0.193 | Sham |  |  |
|  |  |  | 32/57  *p*=0.427 | 19/57  *p*=0.016* | 16/59  *p*=0.001* | 49/58  *p*<0.001* | 41/58  *p*=0.002* | 33/59  *p*=0.435 | 31/59  *p*=0.795 | 25/58  *p=*0.358 | 31/59  *p*=0.795 | 26/58  *p*=0.512 | 7 Hz |  |  |
|  |  |  | 30/58  *p*=0.896 | 33/60  *p*=0.519 | 11/60  *p*<0.001* | 44/60  *p*<0.001* | 48/60  *p*<0.001* | 29/58  *p*=1.000 | 28/60  *p*=0.699 | 16/60  *p*<0.001* | 41/58  *p*=0.002* | 35/59  *p*=0.193 | 18 Hz |  |  |
| 20 | 24 | F | 39/60  *p*=0.018* | 35/59  *p*=0.193 | 19/60  *p*=0.006* | 53/59  *p*<0.001* | 50/60  *p*<0.001* | 34/60  *p*=0.366 | 35/60  *p*=0.245 | 31/58  *p*=0.694 | 44/60  *p*<0.001* | 31/59  *p*=0.897 | Sham |  |  |
|  |  |  | 41/60  *p*=0.006* | 48/60  *p*<0.001* | 19/59  *p*=0.009* | 55/59  *p*<0.001* | 50/59  *p*<0.001* | 33/60  *p*=0.519 | 36/59  *p*=0.117 | 31/59  *p=*0.795 | 36/60  *p*=0.155 | 27/57  *p*=0.791 | 7 Hz |  |  |
|  |  |  | 35/60  *p*=0.245 | 33/59  *p*=0.435 | 14/60  *p*<0.001* | 54/60  *p*<0.001* | 54/60  *p*<0.001* | 31/59  *p*=0.795 | 39/60  *p*=0.027* | 37/60  *p*=0.092 | 32/58  *p*=0.512 | 26/60  *p*=0.366 | 18 Hz |  |  |
| 21 | 23 | F | 29/60  *p*=0.897 | 38/60  *p*=0.052 | 19/60  *p*=0.006* | 40/59  *p*=0.009* | 43/58  *p*<0.001* | 32/59  *p*=0.603 | 35/58  *p*=0.148 | 31/59  *p*=0.795 | 35/60  *p*=0.245 | 38/60  *p*=0.052 | Sham |  |  |
|  |  |  | 29/59  *p*=1.000 | 38/60  *p*=0.052 | 21/60  *p*=0.027* | 47/60  *p*<0.001* | 40/59  *p*=0.009* | 29/60  *p*=0.897 | 31/60  *p*=0.897 | 22/59  *p=*0.067 | 48/57  *p*<0.001* | 37/60  *p*=0.092 | 7 Hz |  |  |
|  |  |  | 38/60  *p*=0.052 | 38/59  *p*=0.036* | 20/58  *p*=0.025* | 54/60  *p*<0.001* | 48/60  *p*<0.001* | 27/58  *p*=0.694 | 34/59  *p*=0.298 | 31/59  *p*=0.795 | 34/59  *p*=0.298 | 34/60  *p*=0.366 | 18 Hz |  |  |
| 22 | 24 | F | 30/58  *p*=0.896 | 29/59  *p*=1.000 | 26/59  *p*=0.435 | 33/58  *p*=0.358 | 30/58  *p*=0.896 | 29/59  *p*=1.000 | 28/59  *p*=0.795 | 34/60  *p*=0.366 | 29/56  *p*=0.894 | 27/58  *p*=0.694 | Sham |  |  |
|  |  |  | 29/59  *p*=1.000 | 33/60  *p*=0.519 | 35/58  *p*=0.148 | 29/58  *p*=1.000 | 27/60  *p*=0.519 | 30/58  *p*=0.896 | 25/59  *p*=0.298 | 31/57  *p=*0.597 | 32/58  *p*=0.512 | 34/59  *p*=0.298 | 7 Hz |  |  |
|  |  |  | 35/60  *p*=0.245 | 30/60  *p*=1.000 | 26/60  *p*=0.366 | 40/59  *p*=0.009* | 34/59  *p*=0.298 | 32/60  *p*=0.699 | 36/60  *p*=0.155 | 32/59  *p*=0.603 | 32/60  *p*=0.699 | 26/60  *p*=0.366 | 18 Hz |  |  |
| 23 | 24 | F | 44/60  *p*<0.001* | 44/59  *p*<0.001* | 9/60  *p*<0.001* | 59/60  *p*<0.001* | 53/60  *p*<0.001* | 33/60  *p*=0.519 | 30/56  *p*=0.689 | 25/60  *p*=0.245 | 47/58  *p*=0.048* | 34/59  *p*=0.298 | Sham |  |  |
|  |  |  | 43/57  *p*<0.001* | 39/57  *p*=0.008* | 18/60  *p*=0.003* | 54/60  *p*<0.001* | 51/59  *p*<0.001* | 28/58  *p*=0.896 | 29/60  *p*=0.897 | 25/58  *p=*0.358 | 36/60  *p*=0.155 | 31/58  *p*=0.694 | 7 Hz |  |  |
|  |  |  | 40/58  *p*=0.005* | 48/58  *p*<0.001* | 7/58  *p*<0.001* | 49/58  *p*<0.001* | 52/58  *p*<0.001* | 32/59  *p*=0.603 | 28/58  *p*=0.896 | 25/59  *p*=0.298 | 35/60  *p*=0.245 | 36/58  *p*=0.087 | 18 Hz |  |  |
| 24 | 22 | F | 33/60  *p*=0.519 | 36/59  *p*=0.117 | 7/60  *p*<0.001* | 54/59  *p*<0.001* | 52/59  *p*<0.001* | 29/60  *p*=0.897 | 28/60  *p*=0.699 | 26/60  *p*=0.366 | 39/59  *p*=0.018* | 39/58  *p*=0.012* | Sham |  |  |
|  |  |  | 34/57  *p*=0.185 | 41/56  *p*=0.001* | 7/60  *p*<0.001* | 58/60  *p*<0.001* | 50/60  *p*<0.001* | 28/60  *p*=0.699 | 25/59  *p*=0.298 | 27/59  *p=*0.603 | 35/59  *p*=0.193 | 32/58  *p*=0.512 | 7 Hz |  |  |
|  |  |  | 32/60  *p*=0.699 | 24/55  *p*=0.419 | 32/57  *p*=0.427 | 33/58  *p*=0.358 | 26/56  *p*=0.689 | 29/59  *p*=1.000 | 33/58  *p*=0.358 | 40/60  *p*=0.013* | 18/56  *p*=0.010* | 22/60  *p*=0.087 | 18 Hz |  |  |
| 25 | 22 | F | 26/59  *p*=0.435 | 28/60  *p*=0.699 | 24/60  *p*=0.155 | 34/60  *p*=0.366 | 34/60  *p*=0.366 | 30/60  *p*=1.000 | 22/59  *p*=0.067 | 29/59  *p*=1.000 | 29/59  *p*=1.000 | 33/59  *p*=0.435 | Sham |  |  |
|  |  |  | 37/60  *p*=0.092 | 22/59  *p*=0.067 | 29/60  *p*=0.897 | 39/59  *p*=0.018* | 35/59  *p*=0.193 | 29/60  *p*=0.897 | 25/60  *p*=0.245 | 28/59  *p=*0.795 | 32/60  *p*=0.699 | 31/60  *p*=0.897 | 7 Hz |  |  |
|  |  |  | 27/60  *p*=0.519 | 26/59  *p*=0.435 | 18/60  *p*=0.003* | 35/60  *p*=0.245 | 36/59  *p*=0.117 | 30/60  *p*=1.000 | 28/60  *p*=0.699 | 31/59  *p*=0.795 | 30/60  *p*=1.000 | 30/60  *p*=1.000 | 18 Hz |  |  |
| 26 | 21 | M | 41/56  *p*=0.001* | 51/60  *p*<0.001* | 11/59  *p*<0.001* | 59/60  *p*<0.001* | 58/59  *p*<0.001* | 34/59  *p*=0.298 | 47/59  *p*<0.001* | 50/57  *p*<0.001* | 37/60  *p*=0.092 | 21/59  *p*=0.036* | Sham |  |  |
|  |  |  | 40/49  *p*<0.001* | 48/56  *p*<0.001* | 11/56  *p*<0.001* | 54/54  *p*<0.001* | 52/54  *p*<0.001* | 35/59  *p*=0.193 | 51/59  *p*<0.001* | 42/59  *p=*0.002* | 34/60  *p*=0.366 | 28/59  *p*=0.795 | 7 Hz |  |  |
|  |  |  | 38/53  *p*=0.002* | 49/57  *p*<0.001* | 11/52  *p*<0.001* | 56/57  *p*<0.001* | 57/59  *p*<0.001* | 37/56  *p*=0.022* | 39/55  *p*=0.003* | 33/54  *p*=0.134 | 31/52  *p*=0.212 | 26/50  *p*=0.888 | 18 Hz |  |  |
| 27 | 29 | F | 32/60  *p*=0.699 | 32/58  *p*=0.512 | 25/60  *p*=0.245 | 29/60  *p*=0.897 | 28/60  *p*=0.699 | 34/59  *p*=0.298 | 36/60  *p*=0.155 | 33/60  *p*=0.519 | 34/60  *p*=0.366 | 31/59  *p*=0.795 | Sham |  |  |
|  |  |  | 39/59  *p*=0.018* | 37/57  *p*=0.033* | 12/57  *p*<0.001* | 49/60  *p*<0.001* | 39/60  *p*=0.027* | 35/60  *p*=0.245 | 36/60  *p*=0.155 | 19/59  *p=*0.009* | 39/60  *p*=0.027* | 36/60  *p*=0.155 | 7 Hz |  |  |
|  |  |  | 46/59  *p*<0.001* | 34/59  *p*=0.298 | 8/60  *p*<0.001* | 57/60  *p*<0.001* | 54/57  *p*<0.001* | 42/59  *p*=0.002* | 40/60  *p*=0.013* | 22/59  *p*=0.067 | 46/59  *p*<0.001* | 36/59  *p*=0.117 | 18 Hz |  |  |
| 28 | 22 | F | 42/58  *p*=0.001* | 41/58  *p*=0.002* | 14/58  *p*<0.001* | 53/57  *p*<0.001* | 55/59  *p*<0.001* | 31/58  *p*=0.694 | 40/58  *p*=0.005* | 41/57  *p*=0.001* | 28/59  *p*=0.795 | 26/57  *p*=0.597 | Sham |  |  |
|  |  |  | 25/60  *p*=0.245 | 32/58  *p*=0.512 | 26/59  *p*=0.435 | 29/60  *p*=0.897 | 24/60  *p*=0.155 | 21/59  *p*=0.036* | 28/59  *p*=0.795 | 28/60  *p=*0.699 | 39/59  *p*=0.018* | 32/59  *p*=0.603 | 7 Hz |  |  |
|  |  |  | 43/60  *p*=0.001* | 45/60  *p*<0.001* | 10/60  *p*<0.001* | 58/60  *p*<0.001* | 57/60  *p*<0.001* | 29/60  *p*=0.897 | 30/60  *p*=1.000 | 34/60  *p*=0.366 | 36/59  *p*=0.117 | 36/60  *p*=0.155 | 18 Hz |  |  |
| 29 | 24 | F | 40/60  *p*=0.013* | 40/60  *p*=0.013* | 28/60  *p*=0.699 | 49/60  *p*<0.001* | 51/60  *p*<0.001* | 35/59  *p*=0.193 | 47/60  *p*<0.001* | 32/60  *p*=0.699 | 43/60  *p*=0.001* | 37/60  *p*=0.092 | Sham |  |  |
|  |  |  | 50/60  *p*<0.001* | 46/60  *p*<0.001* | 11/60  *p*<0.001* | 58/60  *p*<0.001* | 58/60  *p*<0.001* | 33/59  *p*=0.435 | 34/57  *p*=0.185 | 18/57  *p=*0.008* | 42/59  *p*=0.002* | 32/59  *p*=0.603 | 7 Hz |  |  |
|  |  |  | 48/60  *p*<0.001* | 43/60  *p*=0.001* | 24/60  *p*=0.155 | 54/60  *p*<0.001* | 49/60  *p*<0.001* | 27/59  *p*=0.603 | 30/58  *p*=0.896 | 27/58  *p*=0.694 | 38/58  *p*=0.358 | 32/59  *p*=0.603 | 18 Hz |  |  |
| 30 | 24 | F | 53/60  *p*<0.001* | 46/60  *p*<0.001* | 16/60  *p*<0.001* | 54/58  *p*<0.001* | 57/60  *p*<0.001* | 39/60  *p*=0.027* | 38/60  *p*=0.052 | 43/59  *p*=0.001* | 29/60  *p*=0.897 | 19/60  *p*=0.006* | Sham |  |  |
|  |  |  | 49/60  *p*<0.001* | 42/60  *p*=0.003* | 10/60  *p*<0.001* | 59/60  *p*<0.001* | 56/60  *p*<0.001* | 33/59  *p*=0.435 | 40/59  *p*=0.009* | 40/58  *p=*0.005* | 22/59  *p*=0.067 | 27/57  *p*=0.791 | 7 Hz |  |  |
|  |  |  | 45/60  *p*<0.001* | 43/60  *p*=0.001* | 8/60  *p*<0.001* | 59/60  *p*<0.001* | 60/60  *p*<0.001* | 30/60  *p*=1.000 | 24/60  *p*=0.155 | 14/60  *p*<0.001* | 47/60  *p*<0.001* | 40/60  *p*=0.013* | 18 Hz |  |  |
| 31 | 23 | F | 40/60  *p*=0.013* | 47/59  *p*<0.001* | 9/60  *p*<0.001* | 56/60  *p*<0.001* | 55/60  *p*<0.001* | 42/60  *p*=0.003* | 38/59  *p*=0.036* | 16/60  *p*<0.001* | 56/60  *p*<0.001* | 45/58  *p*<0.001* | Sham |  |  |
|  |  |  | 27/54  *p*=1.000 | 45/59  *p*<0.001* | 24/56  *p*=0.350 | 40/57  *p*=0.003* | 48/59  *p*<0.001* | 35/57  *p*=0.111 | 33/50  *p*=0.033* | 11/58  *p*<0.001* | 53/56  *p*<0.001* | 42/54  *p*<0.001* | 7 Hz |  |  |
|  |  |  | 41/60  *p*=0.006* | 48/60  *p*<0.001* | 9/60  *p*<0.001* | 59/60  *p*<0.001* | 57/60  *p*<0.001* | 38/60  *p*=0.052 | 43/60  *p*=0.001* | 14/59  *p*<0.001* | 52/60  *p*<0.001* | 43/60  *p*=0.001* | 18 Hz |  |  |
| 32 | 22 | F | 38/59  *p*=0.036* | 41/59  *p*=0.004* | 15/59  *p*<0.001* | 51/57  *p*<0.001* | 51/58  *p*<0.001* | 34/60  *p*=0.366 | 42/60  *p*=0.003* | 36/57  *p*=0.063 | 31/59  *p*=0.795 | 26/60  *p*=0.366 | Sham |  |  |
|  |  |  | 37/60  *p*=0.092 | 41/60  *p*=0.006* | 12/59  *p*<0.001* | 57/60  *p*<0.001* | 48/60  *p*<0.001* | 33/59  *p*=0.435 | 31/60  *p*=0.897 | 37/60  *p=*0.092 | 30/60  *p*=1.000 | 29/30  *p*<0.001* | 7 Hz |  |  |
|  |  |  | 43/60  *p*=0.001* | 45/59  *p*<0.001* | 9/58  *p*<0.001* | 57/60  *p*<0.001* | 58/60  *p*<0.001* | 37/60  *p*=0.092 | 38/58  *p*=0.025* | 50/59  *p*<0.001* | 23/57  *p*=0.185 | 18/58  *p*=0.005* | 18 Hz |  |  |
| 33 | 25 | F | 47/60  *p*<0.001* | 51/60  *p*<0.001* | 11/59  *p*<0.001* | 59/60  *p*<0.001* | 57/60  *p*<0.001* | 35/60  *p*=0.245 | 43/60  *p*=0.001* | 24/60  *p*=0.155 | 44/60  *p*<0.001* | 45/58  *p*<0.001* | Sham |  |  |
|  |  |  | 42/60  *p*=0.003* | 54/59  *p*<0.001* | 12/60  *p*<0.001* | 60/60  *p*<0.001* | 56/60  *p*<0.001* | 29/60  *p*=0.897 | 36/60  *p*=0.155 | 25/60  *p=*0.245 | 48/60  *p*<0.001* | 42/60  *p*=0.003* | 7 Hz |  |  |
|  |  |  | 43/58  *p*<0.001* | 48/58  *p*<0.001* | 23/58  *p*=0.148 | 55/59  *p*<0.001* | 56/59  *p*<0.001* | 33/59  *p*=0.435 | 33/59  *p*=0.435 | 22/59  *p*=0.067 | 45/60  *p*<0.001* | 36/59  *p*=0.117 | 18 Hz |  |  |
| 34 | 26 | F | 45/59  *p*<0.001* | 40/59  *p*=0.009* | 12/59  *p*<0.001* | 56/59  *p*<0.001* | 56/59  *p*<0.001* | 48/60  *p*<0.001* | 46/60  *p*<0.001* | 17/59  *p*=0.002* | 56/59  *p*<0.001* | 53/60  *p*<0.001* | Sham |  |  |
|  |  |  | 41/59  *p*=0.004* | 41/58  *p*=0.003* | 13/56  *p*<0.001* | 51/57  *p*<0.001* | 47/56  *p*<0.001* | 41/60  *p*=0.006* | 35/60  *p*=0.245 | 18/60  *p=*0.003* | 47/60  *p*<0.001* | 51/60  *p*<0.001* | 7 Hz |  |  |
|  |  |  | 47/60  *p*<0.001* | 45/60  *p*<0.001* | 15/60  *p*<0.001* | 56/60  *p*<0.001* | 51/60  *p*<0.001* | 43/60  *p*=0.001* | 43/60  *p*=0.001* | 25/60  *p*=0.245 | 58/60  *p*<0.001* | 47/60  *p*<0.001* | 18 Hz |  |  |
| **Overall** | | | **67.58 ± 12.77%**  ***t*_33_ = 8.026**  ***p* < .001***  ***d* = 1.377** | **68.34 ± 12.67%**  ***t*_33_ = 8.440**  ***p* < .001***  ***d* = 1.447** | **29.40 ± 16.70%**  ***t*_33_ = -7.194**  ***p* < .001***  ***d* = 1.234** | **84.15 ± 16.37%**  **Z = 1.124**  ***p* < .001***  ***d* = 2.086** | **80.38 ± 17.70%**  **Z = 1.095**  ***p* < .001***  ***d* = 1.716** | **59.72 ± 10.27%**  **Z = 1.035**  ***p* < .001***  ***d* = 0.947** | **64.57 ± 12.87%**  ***t*_33_ = 6.605**  ***p* < .001***  ***d* = 1.133** | **53.86 ± 23.73%**  ***t*_33_ = 0.942**  ***p* = .353**  ***d* = 0.162** | **65.93 ± 18.67%**  ***t*_33_ = 4.977**  ***p* < .001***  ***d* = 0.854** | **54.51 ± 16.85%**  ***t*_33_ = 1.560**  ***p* = .128**  ***d* = 0.268** | **Sham** |  |  |
|  | | | **65.81 ± 11.88%**  ***t*_33_ = 7.757**  ***p* < .001***  ***d* = 1.330** | **67.22 ± 13.86%**  ***t*_33_ = 7.245**  ***p* < .001***  ***d* = 1.243** | **29.33 ± 19.40%**  **Z = 0.933**  ***p* < .001***  ***d* = 1.065** | **84.28 ± 17.91%**  **Z = 1.084**  ***p* < .001***  ***d* = 1.914** | **78.81 ± 18.44%**  **Z = 1.067**  ***p* < .001***  ***d* = 1.563** | **58.10 ± 9.76%**  **Z = 0.928**  ***p* < .001***  ***d* = 0.830** | **61.64 ± 12.67%**  ***t*_33_ = 5.355**  ***p* < .001***  ***d* = 0.918** | **52.59 ± 21.63%**  ***t*_33_ = 0.698**  ***p* = .490**  ***d* = 0.120** | **63.29 ± 18.72%**  ***t*_33_ = 4.140**  ***p* < .001***  ***d* = 0.710** | **57.88 ± 18.88%**  ***t*_33_ = 2.433**  ***p* = .021***  ***d* = 0.417** | **7 Hz** |  |  |
|  | | | **68.11 ± 10.83%**  **Z = 1.094**  ***p* < .001***  ***d* = 1.673** | **68.80 ± 12.48%**  ***t*_33_ = 8.784**  ***p* < .001***  ***d* = 1.506** | **29.22 ± 18.84%**  **Z = 0.955**  ***p* < .001***  ***d* = 1.103** | **85.80 ± 16.36%**  **Z = 1.117**  ***p* < .001***  ***d* = 2.186** | **82.05 ± 17.93%**  **Z = 1.097**  ***p* < .001***  ***d* = 1.787** | **60.02 ± 10.47%**  ***t*_33_ = 5.581**  ***p* < .001***  ***d* = 0.957** | **62.01 ± 11.31%**  ***t*_33_ = 6.189**  ***p* < .001***  ***d* = 1.061** | **51.37 ± 23.06%**  ***t*_33_ = 0.348**  ***p* = .730**  ***d* = 0.060** | **65.00 ± 20.53%**  ***t*_33_ = 4.260**  ***p* = < .001***  ***d* = 0.731** | **55.55 ± 16.59%**  ***t*_33_ = 1.951**  ***p* = .060**  ***d* = 0.335** | **18 Hz** |  |  |

**Figure S2.** Comparison of the performances in the two discrimination tasks in all five types of trials considering only the sham condition. Boxplots represent median, first quartile, third quartile, ranges and outliers. The x-axis refers to the different types of trials (control ratios and illusory tests). The y-axis refers to the estimated probability (obtained through the effects package) of correct responses (i.e., choices for the larger target stimulus and numerosity in control trials and the choices for the stimulus and the numerosity presented in the small context in illusory trials).

**
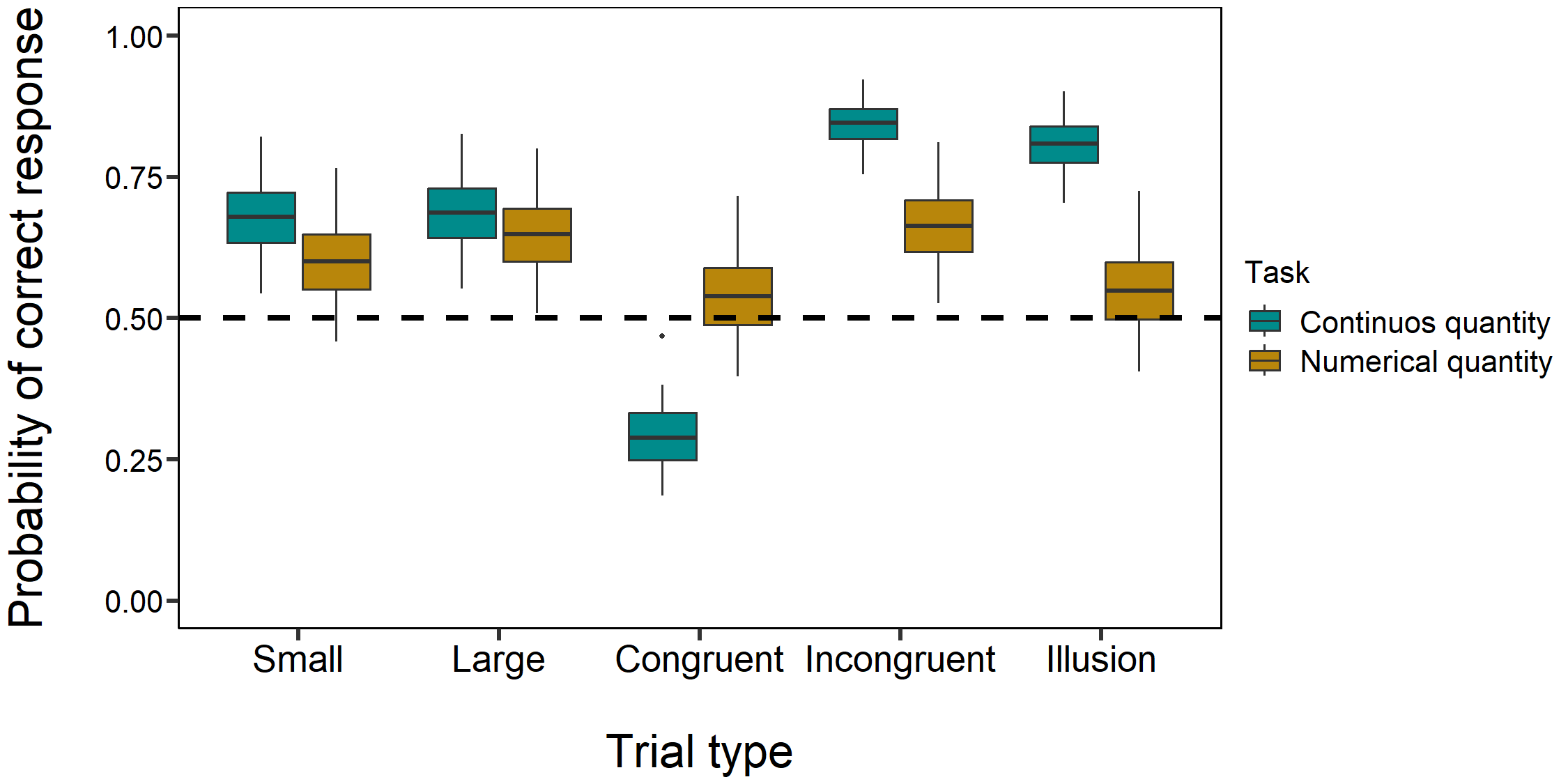
**

**Figure S3.** Comparison of the performances in the two discrimination tasks in all five types of trials considering only the 7Hz stimulation. Boxplots represent median, first quartile, third quartile, ranges and outliers. The x-axis refers to the different types of trials (control ratios and illusory tests). The y-axis refers to the estimated probability (obtained through the effects package) of correct responses (i.e., choices for the larger target stimulus and numerosity in control trials and the choices for the stimulus and the numerosity presented in the small context in illusory trials).

**
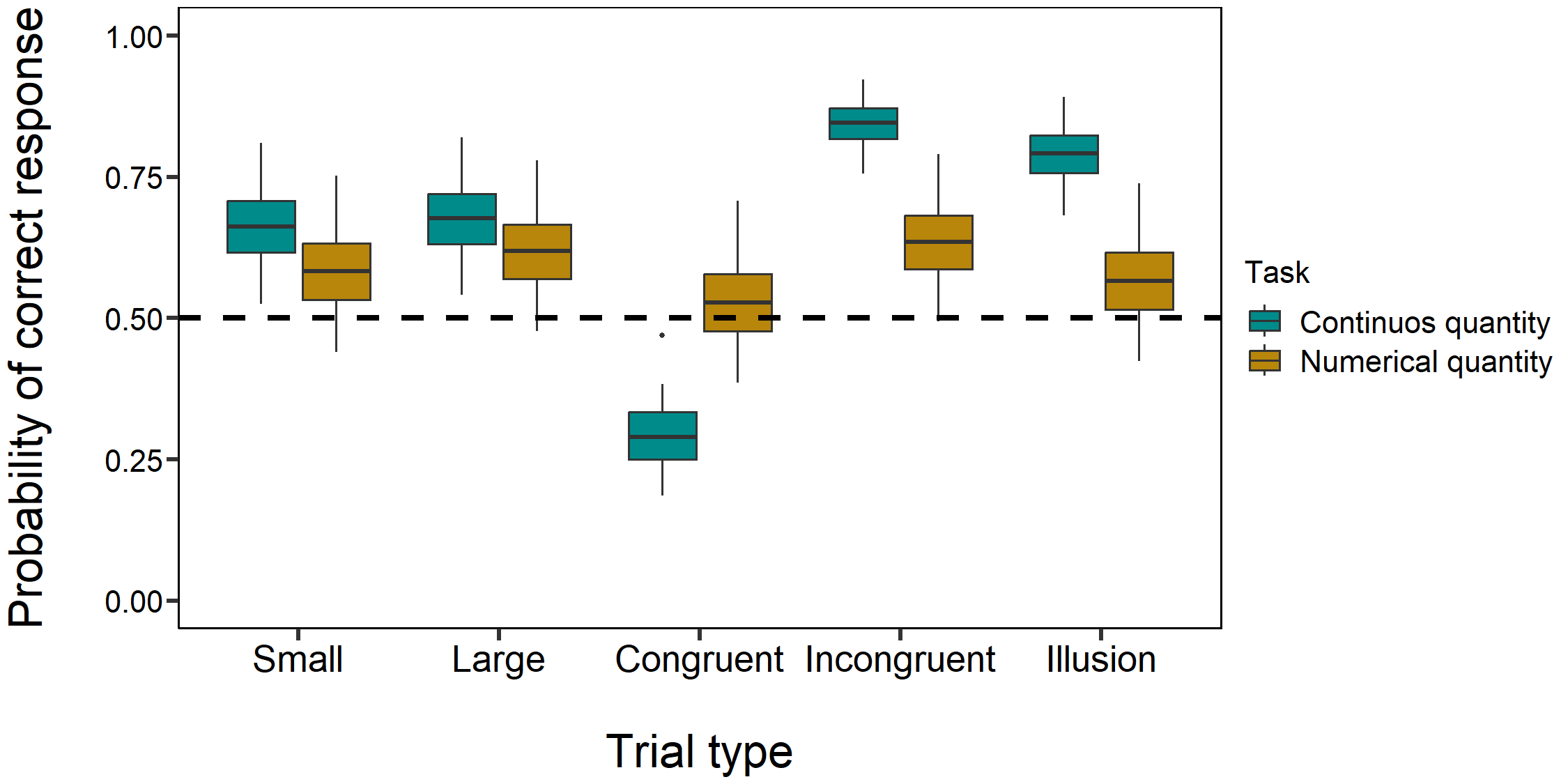
**

**Figure S4.** Comparison of the performances in the two discrimination tasks in all five types of trials considering only the 18Hz stimulation. Boxplots represent median, first quartile, third quartile, ranges and outliers. The x-axis refers to the different types of trials (control ratios and illusory tests). The y-axis refers to the estimated probability (obtained through the effects package) of correct responses (i.e., choices for the larger target stimulus and numerosity in control trials and the choices for the stimulus and the numerosity presented in the small context in illusory trials).

**
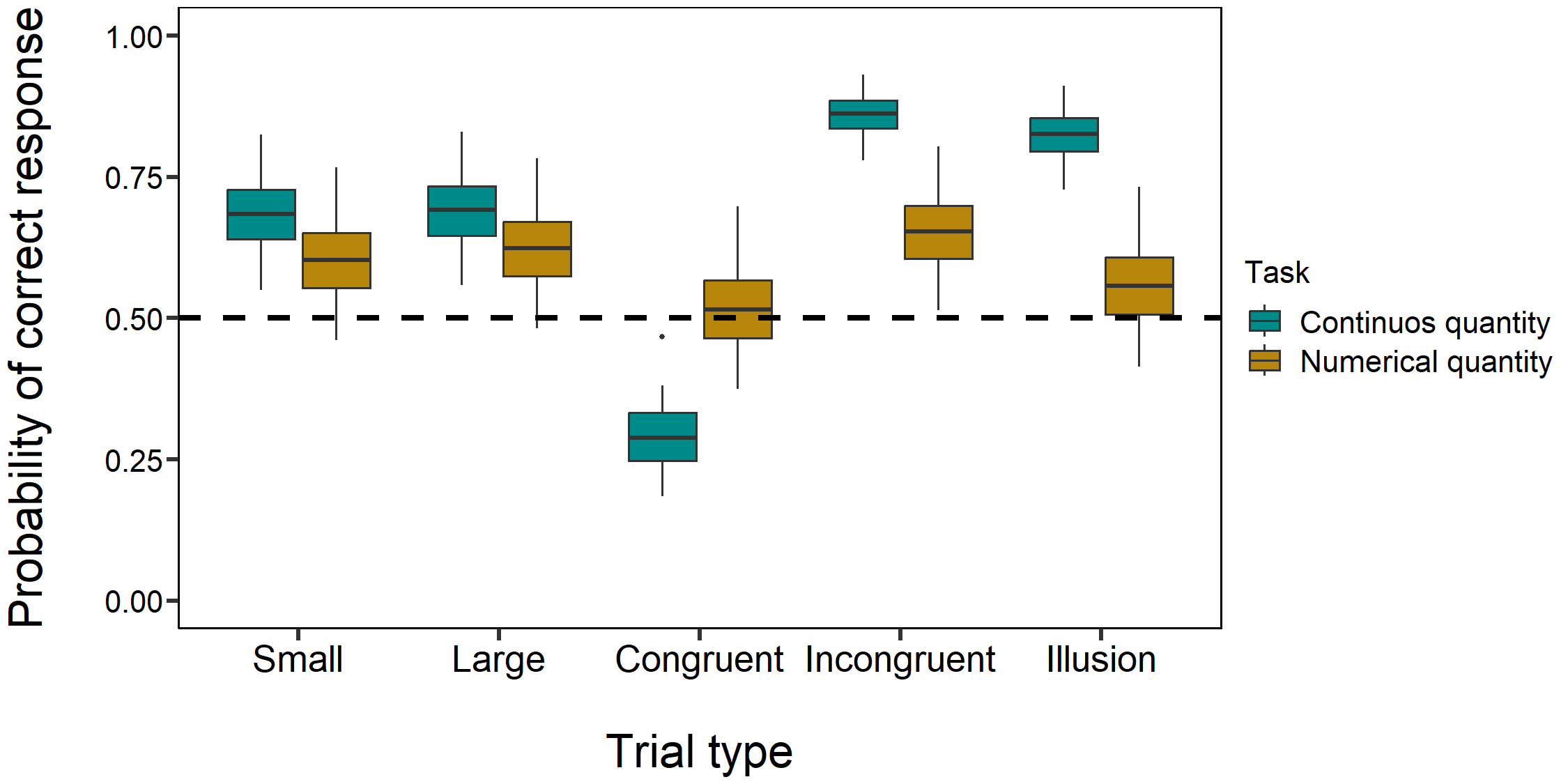
**

**Table S2.** Post-hoc comparisons of all the GLMMs.

|  | **Comparison** | **OR** | **Lower CI** | **Upper CI** | **SE** | ***z*** | ***p* adjusted** |
| --- | --- | --- | --- | --- | --- | --- | --- |
| **Model on numerical discrimination** | congruent / incongruent | 0.600 | 0.539 | 0.667 | 0.023 | -13.524 | < 0.001 * |
|  | congruent / large | 0.655 | 0.590 | 0.728 | 0.025 | -11.245 | < 0.001 * |
|  | congruent / small | 0.759 | 0.684 | 0.842 | 0.028 | -7.411 | < 0.001 * |
|  | congruent / illusion | 0.887 | 0.799 | 0.984 | 0.033 | -3.233 | 0.001 * |
|  | incongruent / large | 1.093 | 0.981 | 1.217 | 0.042 | 2.312 | 0.021 * |
|  | incongruent / small | 1.265 | 1.137 | 1.408 | 0.048 | 6.181 | < 0.001 * |
|  | incongruent / illusion | 1.479 | 1.330 | 1.645 | 0.056 | 10.328 | < 0.001 * |
|  | large / small | 1.158 | 1.041 | 1.287 | 0.044 | 3.872 | < 0.001 * |
|  | large / illusion | 1.353 | 1.218 | 1.504 | 0.051 | 8.035 | < 0.001 * |
|  | small / illusion | 1.169 | 1.053 | 1.298 | 0.044 | 4.180 | < 0.001 * |
| **Model on continuous quantity discrimination** | congruent / incongruent | 0.068 | 0.060 | 0.078 | 0.003 | -57.449 | < 0.001 * |
|  | congruent / large | 0.182 | 0.162 | 0.204 | 0.007 | -41.927 | < 0.001 * |
|  | congruent / small | 0.190 | 0.169 | 0.213 | 0.008 | -41.084 | < 0.001 * |
|  | congruent / illusion | 0.092 | 0.082 | 0.105 | 0.004 | -53.858 | < 0.001 * |
|  | incongruent / large | 2.668 | 2.344 | 3.037 | 0.123 | 21.283 | < 0.001 * |
|  | incongruent / small | 2.787 | 2.450 | 3.171 | 0.128 | 22.322 | < 0.001 * |
|  | incongruent / illusion | 1.356 | 1.181 | 1.556 | 0.067 | 6.197 | < 0.001 * |
|  | large / small | 1.045 | 0.934 | 1.168 | 0.042 | 1.096 | 0.273 |
|  | large / illusion | 0.508 | 0.450 | 0.574 | 0.022 | -15.531 | < 0.001 * |
|  | small / illusion | 0.486 | 0.431 | 0.549 | 0.021 | -16.611 | < 0.001 * |
|  | 18 hz / 7 hz | 1.111 | 1.024 | 1.206 | 0.038 | 3.080 | < 0.01 * |
|  | 18 hz / control | 1.061 | 0.978 | 1.152 | 0.036 | 1.732 | 0.125 |
|  | 7 hz / control | 0.955 | 0.881 | 1.035 | 0.032 | -1.363 | 0.173 |
| **Overall model: comparison between numerical and continuous quantity discrimination** | congruent / incongruent | 0.206 | 0.189 | 0.224 | 0.006 | -52.897 | < 0.001 * |
|  | congruent / large | 0.349 | 0.323 | 0.377 | 0.010 | -38.104 | < 0.001 * |
|  | congruent / small | 0.384 | 0.356 | 0.415 | 0.011 | -34.937 | < 0.001 * |
|  | congruent / illusion | 0.291 | 0.268 | 0.315 | 0.008 | -43.033 | < 0.001 * |
|  | incongruent / large | 1.697 | 1.56 | 1.846 | 0.051 | 17.671 | < 0.001 * |
|  | incongruent / small | 1.867 | 1.718 | 2.030 | 0.056 | 20.999 | < 0.001 * |
|  | incongruent / illusion | 1.414 | 1.296 | 1.542 | 0.044 | 11.200 | < 0.001 * |
|  | large / small | 1.100 | 1.019 | 1.188 | 0.030 | 3.483 | < 0.001 * |
|  | large / illusion | 0.833 | 0.769 | 0.903 | 0.024 | -6.350 | < 0.001 * |
|  | small / illusion | 0.757 | 0.699 | 0.820 | 0.022 | -9.745 | < 0.001 * |
|  | 18 Hz / 7 Hz | 1.063 | 1.008 | 1.122 | 0.024 | 2.729 | 0.019 * |
|  | 18 Hz / control | 1.009 | 0.956 | 1.065 | 0.023 | 0.388 | 0.698 |
|  | 7 Hz / control | 0.949 | 0.900 | 1.001 | 0.021 | -2.358 | 0.028 * |
|  | Continuous quantity / numerical discrimination | 1.469 | 1.418 | 1.523 | 0.027 | 21.065 | < 0.001 * |
|  | Continuous quantity:  congruent / incongruent | 0.071 | 0.062 | 0.081 | 0.003 | -57.23 | < 0.001 * |
|  | Continuous quantity:  congruent / large | 0.187 | 0.165 | 0.211 | 0.008 | -41.701 | < 0.001 * |
|  | Continuous quantity:  congruent / small | 0.195 | 0.173 | 0.220 | 0.008 | -40.802 | < 0.001 * |
|  | Continuous quantity:  congruent / illusion | 0.096 | 0.084 | 0.109 | 0.004 | -53.69 | < 0.001 * |
|  | Continuous quantity:  incongruent / large | 2.636 | 2.295 | 3.028 | 0.121 | 21.162 | < 0.001 * |
|  | Continuous quantity:  incongruent / small | 2.752 | 2.398 | 3.159 | 0.126 | 22.194 | < 0.001 * |
|  | Continuous quantity:  incongruent / illusion | 1.351 | 1.165 | 1.566 | 0.066 | 6.159 | < 0.001 * |
|  | Continuous quantity:  large / small | 1.044 | 0.926 | 1.177 | 0.041 | 1.088 | 0.277 |
|  | Continuous quantity:  large / illusion | 0.512 | 0.450 | 0.584 | 0.022 | -15.462 | < 0.001 * |
|  | Continuous quantity:  small / illusion | 0.491 | 0.431 | 0.559 | 0.021 | -16.513 | < 0.001 * |
|  | Numerical:  congruent / incongruent | 0.599 | 0.534 | 0.671 | 0.023 | -13.537 | < 0.001 * |
|  | Numerical:  congruent / large | 0.654 | 0.584 | 0.733 | 0.025 | -11.27 | < 0.001 * |
|  | Numerical:  congruent / small | 0.758 | 0.677 | 0.849 | 0.028 | -7.408 | < 0.001 * |
|  | Numerical:  congruent / illusion | 0.886 | 0.792 | 0.991 | 0.033 | -3.255 | 0.001 * |
|  | Numerical:  incongruent / large | 1.093 | 0.973 | 1.227 | 0.042 | 2.303 | 0.022 * |
|  | Numerical:  incongruent / small | 1.267 | 1.129 | 1.421 | 0.048 | 6.208 | < 0.001 * |
|  | Numerical:  incongruent / illusion | 1.480 | 1.320 | 1.660 | 0.056 | 10.332 | < 0.001 * |
|  | Numerical:  large / small | 1.159 | 1.034 | 1.300 | 0.044 | 3.902 | < 0.001 * |
|  | Numerical:  large / illusion | 1.355 | 1.209 | 1.519 | 0.051 | 8.051 | < 0.001 * |
|  | Numerical:  small / illusion | 1.169 | 1.044 | 1.308 | 0.044 | 4.166 | < 0.001 * |
